# "When patients have cancer, they stop seeing me" – the role of the general practitioner in early follow-up of patients with cancer – a qualitative study

**DOI:** 10.1186/1471-2296-7-19

**Published:** 2006-03-21

**Authors:** Tor Anvik, Knut A Holtedahl, Hege Mikalsen

**Affiliations:** 1 Institute of Community Medicine, University of Tromsø, N-9037 Tromsø, Norway; 2 Department of Gastrointestinal Surgery, University Hospital of Northern Norway, N-9038 Tromsø, Norway

## Abstract

**Background:**

The role of the general practitioner (GP) in cancer follow-up is poorly defined. We wanted to describe and analyse the role of the GP during initial follow-up of patients with recently treated cancer, from the perspective of patients, their relatives and their GPs.

**Methods:**

One focus group interview with six GPs from the city of Bodø and individual interviews with 17 GPs from the city of Tromsø in North Norway. Text analysis of the transcribed interviews and of free text comments in two questionnaires from 91 patients with cancer diagnosed between October 1999 and September 2000 and their relatives from Tromsø.

**Results:**

The role of the GP in follow-up of patients with recently treated cancer is discussed under five main headings: *patient involvement, treating the cancer and treating the patient, time and accessibility, limits to competence*, and *the GP and the hospital should work together*.

**Conclusion:**

The GP has a place in the follow-up of many patients with cancer, also in the initial phase after treatment. Patients trust their GP to provide competent care, especially when they have more complex health care needs on top of their cancer. GPs agree to take a more prominent role for cancer patients, provided there is good access to specialist advice. Plans for follow-up of individual patients could in many cases improve care and cooperation. Such plans could be made preferably before discharge from in-patient care by a team consisting of the patient, a carer, a hospital specialist and a general practitioner. Patients and GPs call on hospital doctors to initiate such collaboration.

## Background

General practitioners (GPs) participate in the follow-up of patients with cancer [1], but the role of the GP is poorly defined and varies between different places and for different kinds of patients. Research on the role of the GP in follow up of patients with cancer has suggested that improved information giving from the hospital may increase the GP's ability to determine the patients' need for support [2] and facilitate cooperation between the hospital specialists and the GP [3]. In one study three out of four GPs stated that they were ready to take shared responsibility for follow up of the patients [4]. However, we have found no reports from the last 30 years giving a more comprehensive description of the role of the GP in follow up for patients during the first months or years after diagnosis.

We previously performed a randomized controlled trial with 91 patients recently treated for different forms of cancer [5]. They were asked to complete two self-administered questionnaires dealing with quality of life (EORTC QLQ C-30) and satisfaction with care (Patients' Views on Cancer Services). After randomisation, 41 intervention patients were offered two extra consultations with and increased accessibility to their GP and 50 control patients received no extra follow-up. After six months the only difference concerning quality of life or patient satisfaction with care was increased satisfaction with care on the part of relatives of intervention patients. However, free text comments from patients in the questionnaires suggested that GP participation might nevertheless be desirable in many cases. We therefore performed another study in order to look deeper into the role of the GP in follow-up for patients with recently treated cancer. Our goal here is to report, explore and interpret statements from GPs about their role in such follow up and to compare their statements with written comments from patients and their relatives.

The municipality of Tromsø introduced a personal list system with GPs for all inhabitants from 1993. The system was implemented in the rest of the nation in 2001. The GP traditionally has the gatekeeper role in that patients in most cases must see their GP before seeing a specialist or going to a hospital. Patients with cancer are diagnosed and treated mainly by specialists in hospitals and subsequently as outpatients in the same hospital. Most initial and even long-term follow-up also takes place in the hospital outpatient clinic.

## Methods

In order to ensure richness and variance in our material and validity in our findings we included statements from different groups with different perspectives on the same topic. With this qualitative approach we hoped to get insight into people's ideas and the reasons for their actions [6]. Our material came from five sources (Table 1):

**Table 1:** Collection of data

Who	Method	Type of text	Number of words
6 GPs from another town	Focus group discussion about GP role in cancer follow-up (taped)	Transcribed discussion	14598
13 GPs with at least two randomized patients	Individual interviews with the GPs (taped)	Transcribed interviews	86195
4 GPs who had at least one eligible patient who died before finishing treatment	Individual interviews with the four GPs (taped)	Transcribed interviews	3169
91 randomized patients 45 relatives 1 friend	Self-administered questionnaires at start and after six months One page enclosed for relative or friend	Written comments transcribed from the patients and their relatives and friends	16255 from patients 838 from relatives and friends
41 intervention patients	Invited consultation with the personal GP at start and after six months Three open-ended questions asked by the GP	Patients' answers written down by the GP and transcribed	16538

**Figure 1:**
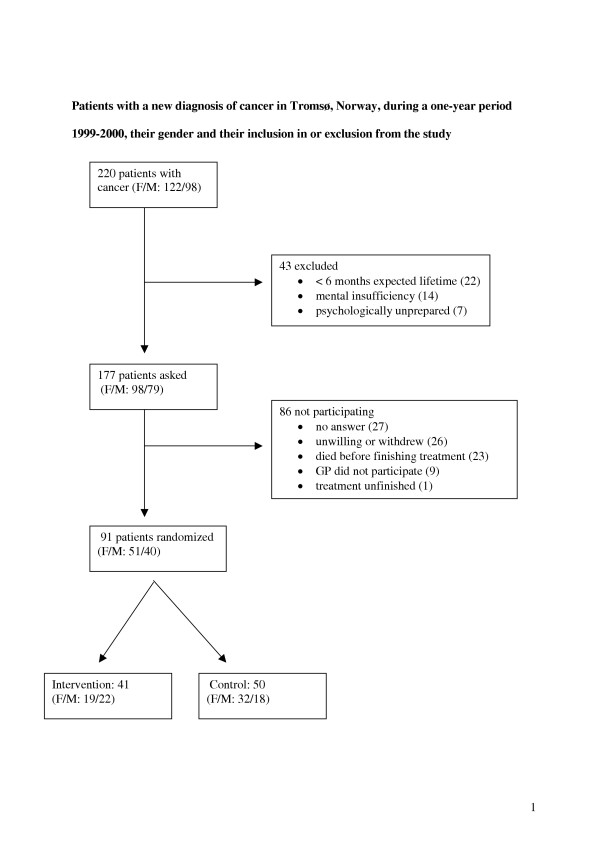
Patients with a new diagnosis of cancer in Tromsø, Norway, during a one-year period 1999–2000, their gender and their inclusion in or exclusion from the study.

1. Verbatim transcription of a focus group interview with six GPs (three females) from the North Norwegian town of Bodø in 2003, focusing on their own experience with patients with recently diagnosed cancer and asking their opinion about the role of the GP for these patients

2. Verbatim transcription of semi-structured interviews with 13 GPs (four females) in 2003 who had at least two patients among the 91 included intervention and control patients from Tromsø in North Norway

3. Verbatim transcription of semi-structured interviews with four GPs (one female) in 2002 whose patients were among the 91 included in the study and who died before finishing treatment. The GPs were interviewed about their role for these patients after diagnosis

4. Free text comments from self-administered questionnaires in 2000 and 2001 from the 91 included patients and from their relatives and friends (Figure 1)

5. Comments written down by the GPs in 2000 and 2001 when interviewing the 41 patients in the intervention group at the start of the study and six months later. The GPs asked three questions concerning quality of life, impact from the cancer disease on daily life and information received about prospects for cure:

-"Everything considered, how is your life?"

-"How does the cancer disease influence your life?"

-"Following hospital treatment, what information did you get about your prospects for cure?" (during the first interview only) [5].

Quantitatively, the interviews with the 13 GPs constitute the main body of the text material. The comments from patients are important to complete the picture and quotations from both sources are presented in the article. The interviews started by asking the GPs to summarize the medical files for each included patient from the time when the diagnosis of cancer had been made. We proceeded with questions about the actions taken by the GP and the cooperation with hospitals, specialists and other health personnel. The main parts of the interviews focused on whether and how the follow-up for the particular patient might be improved and how the GPs viewed their role in follow up of each of the patients. These interviews lasted from one to two-and-a-half hours and involved from one to five patient records. All of the 13 GPs had patients in the intervention group as well as in the control group.

In order to uncover and report how the informants in their own words understood the role, intentions, actions and attitudes of the GPs we adopted a phenomenological approach to interpret the text [7-9]. The transcribed text was entered for analysis using computer-based software QSR-N6 (NUD*IST, La Trobe University, 2002) [10]. We used sentences for text units in N6 and marked and coded text units that we found significant when reading the text repeatedly. The "spread selection" option in QSR-N6 made it possible to mark and code multiple sentences when more than one sentence in the same paragraph contained significant text on the same topic. Sentences from different documents and with the same codings were viewed together using the "node explorer" in QSR-N6. These codings (named 'nodes' in N6) were analysed and aggregated into thematic categories and then restructured in order to create broader themes that were given new codes. These broader themes were revised after repeated analysis of the text and discussion between the authors, until we found no new or additional themes and felt that most central themes had been identified. We mailed a copy of the draft manuscript to all GPs who had been interviewed and received some comments that have been taken into account.

All included patients gave written consent to participate in the study. The Regional Committee of Medical Research Ethics in Northern Norway approved the study and The Norwegian Data Inspectorate gave permission to create a patient register. The Norwegian Directorate of Health granted the author KH access to patient data.

## Results

We identified five main themes:

### 1. Patient involvement

Patients differed as to how much they preferred to be involved in information and choice of treatment. Many patients emphasised the need to be fully informed about the disease and to be involved in plans for investigation and treatment and stated that hospital doctors should be more active in eliciting the views of the patients.

"I am the kind of person who asks a lot of questions, and therefore I get enough answers... None of the doctors ever asked, 'Is there anything you wonder about or want to ask about?"' P30, woman, 55, with breast cancer

"I also find it a big problem that I as a patient was not invited to take part in the treatment; that everything was decided on beforehand; I was not allowed to take my share of the responsibility for my own treatment. I believe that this is important for the healing process." P54, woman, 44, with cancer of the uterine cervix

Some patients preferred not to talk much about the cancer, were happy to leave decisions to the doctor and did not want their relatives or friends to ask them questions about their cancer. Many of these patients wanted to decide for themselves when and how to talk to other people about their own views and feelings.

"She has in a way put the disease behind her and wants to look ahead. She is fed up with other people nagging about "how are you" and the children always asking "are you sure you do not feel anything wrong?"" P87, woman, 76, with cancer of the ovaries, written down by her GP

"As a patient one does not have the knowledge to participate in decisions". P64, man, 44, with lymphoma

"The patient has managed on her own and there has been no need for care from me. We are two and we talk and you might say that this is some kind of therapy". Spouse of P03, man, 82, with prostate cancer

GPs were aware that patients differ in this respect and gave examples of their own flexibility. Some of the GPs described how they had clarified what the individual patient preferred, respected the patient's wish and acknowledged the patient's behaviour as a positive way of coping with cancer. Many were critical towards general guidelines for how patients with cancer should be handled and emphasized that all patients should be treated individually.

"Different patients have different needs.... Having one fixed policy – we do it this or that way – I would feel that to be wrong" GP 16

"I might have talked more to her. As for Tom, he would have found it very annoying. ---All in all I believe you have to go at it individually, you must try to deal with the patient at hand" GP 10 about P41, woman, 88, with rectal cancer, and P46, man, 75, with colon cancer

"Yes, I will describe him as a closed, introvert person who denied much about his cancer, he indicated that this was his way of managing it" GP 12 about P60, man, 66, with rectal cancer

### 2. Treating the cancer and treating the patient

Most patients and GPs stated that treatment of cancer is best taken care of in hospital. However, many patients and GPs stated that the GP should participate in taking care of the patient as a whole person. The general condition, pain, anxiety, family problems or economy may present more pressing problems than the cancer itself, and hospital doctors were not expected to deal with other problems than those connected to the cancer.

"What I miss the most during the care (in the hospital) is that I was not treated as a whole person. My view is that the focus was on the cancer and that the therapy and the consequences of therapy were not seen in connection with the physical problems I already had from previous cancer treatment." P54, woman, 44, with cancer of the uterine cervix

"A good GP is decisive for follow-up. In my case, other things happened after my operation. An old inflammation in my arm, hypertension, fatigue and reduced capacity for work. I also got climacteric problems because I had to stop taking the pills I have taken for ten years. I have been lucky to have a GP who has taken care of me in a good way." P09, woman, 55, with breast cancer

"I have a GP who, in spite of busy days, sees me as a person. I am very satisfied with him." P40, woman, 45, with breast cancer

"Follow-up for the cancer has in my opinion been the responsibility of the hospital, because they have made these investigations. Follow-up for the consequences of the cancer I feel has been my job" GP 10 about P40, woman, 45, with breast cancer

"The situation as a whole is not good. She is often dizzy, is bothered by a feeling of not fulfilling her household duties (she is aware of what is expected of her as a "housewife"), and she is bothered by her stoma when in public" Son of P41, woman, 86, with rectal cancer

### 3. Time and accessibility

Many patients reported time constraints and lack of continuity in follow-up from hospital doctors. Some patients had visited the outpatient clinic a number of times without seeing the same doctor twice.

"The meetings with doctors during follow-up consultations (in the hospital) are difficult. Never the same doctor. Busy they are, and superficial is my feeling about the situation. The GP is indispensable." P61, woman, 60, with breast cancer

"At the start of the treatment my biggest problem was that I doubted that the doctors had sufficient knowledge about my kind of cancer...little time was found and I got the impression that much of the time was spent reading my medical record. I saw four different doctors the four first times" P85, man, 67, with lymphoma

All the GPs acknowledged the importance of being accessible to patients with cancer and had various ways of organising this. Some GPs saw follow-up of patients with cancer as demanding extra time but said that this was not a big problem because there are never many such patients at the same time on one GP's list.

"I would like to have a specialist in the hospital to telephone. If I were to undertake follow-up for this patient I would certainly have to spend a lot of time making telephone calls trying to find the right person to talk to at the hospital. I sometimes feel that this takes a lot of time." GP 03 talking about P03, man, 82, with prostate cancer

"Some may feel that this is a little troublesome or complicated, and not to mention time demanding, but as GPs we do not have that many at the same time". GP-A in focus group

"I have found a good way for attending to these patients; if there may be a problem with time I often plan a visit, say two weeks ahead, and then it can be altered as needed, but I have found that it relieves some of the time pressure". GP-C in focus group

"In the end I gave him my home phone number, but he never called. It's sort of like having tranquillisers in your pocket but never touching them. You know they're there, and that's it". GP-D in focus group

### 4. Limits to competence

Factual knowledge is important, but continuity and communication skills are also important parts of GP competence. Several patients stated that they trust their GP to take good care of them and their cancer, provided they know their patients well enough. Two patients did not feel safe with their personal GP; one of them saw the GP once more and talked it through with him and ended up with trusting the GP, the other chose another GP.

"I have been happy to have a very competent personal GP who has supported me in a great way" P09, woman, 55, with breast cancer

"I feel that my GP has taken good care of me and taken me seriously." P26, woman, 62, with breast cancer

"One month after the operation I went to see my GP only to talk to him, tell him in a polite way how (disturbed) I had felt then, and many times before. He thanked me for coming, and after that I feel that my GP takes my problems and worries seriously." P42, woman, 55, with rectal cancer

None of the GPs in our study had participated in administering cytostatic treatment. However, almost all had participated in treatment of patients with advanced stages of cancer where palliation and/or care at the end of life were a subject. Many GPs said that they felt comfortable with taking responsibility for such treatment and had found ways of obtaining the requisite knowledge from the specialists when they needed it. These GPs did not feel that an initial lack of factual knowledge should deter them from engaging in treatment and follow-up when the patients wanted it.

"The time space between each cancer case makes it impossible to keep up with all new knowledge, at least for me". GP 02

"I am a young doctor, so I frequently call the specialist to check out things I wonder about...one can always phone the outpatient specialist if one wants, or talk to a haematologist." GP 06 about P62, man, 65, with lymphoma

"I could talk to anyone of them (oncologists in the hospital), they took one step back and discussed it between themselves, and then gave me advice about medication...Obviously, they have a unique knowledge in this field that I do not have. The medication I gave could not have been so accurate if it were not for them, that's for sure." GP-B in focus group

GPs who had been involved in the care of patients with cancer found that this had been rewarding and stated that it had heightened their job satisfaction. Care for such patients may become an opportunity for the GP to make a difference for seriously ill patients.

"The fact that they (the patient and his family) told me they felt cared for, that was nice. It gives you satisfaction as a GP when you feel you can do something good... and even that you can support them by the end of life; that was really nice...Taking care of people – that's what it's all about, isn't it?" GP06 about P12, woman, 57, with oral cancer

"I found this a very meaningful way of working as a doctor". GP-A from focus group

### 5. The GP and the specialist should work together

Ideally, primary and secondary cares reinforce each other. There is an increasing trend towards patients being treated and followed in the hospital outpatient clinics rather than as in-patients, and some outpatient services might conceivably be delegated to primary care. Also, increasing attention towards good palliative care may create an opportunity for closer collaboration between GPs and specialists. A new diagnosis is an event that may increase the need for co-ordination, but patients with cancer on the contrary often disappear from the GPs attention.

"He has had no need to consult (his GP) because he has regular follow-up at the hospital. He feels safe about treatment schemes and follow-up." GP 21 about P69, man, 78, with laryngeal cancer

"I expect that I am not the only one who has had problems with well-being after cancer surgery. The hospital should encourage the patients to contact their GP for further support". P09, woman, 56, with breast cancer – reported by her GP

A number of GPs suggested that care for patients with cancer could be improved if the specialist invited the patient, the family and the personal GP to a meeting when the patient is ready for discharge from in-patient care, or when therapy is finished. The main goals for such a meeting could be to share information and define roles in further follow-up.

"It would have been nice if the patient, the surgeon and I could have met and talked about how to do things in the near future." GP 01 about P05, man, 66, with prostate cancer

"It usually works better to cooperate if you have met with people and seen them; and the patient can see that this is teamwork... This is done a lot in psychiatry, they invite us to meetings when a patient is ready to leave the hospital; so we are quite used to attending such meetings". GP 12 about P79, woman, 52, with breast cancer

"I participated in a responsibility group. That is a model I believe in. We all met in the patient's home. I experienced this as something very positive, everybody had a genuine interest in co-operation." GP-B in focus group

## Discussion

### The methods

We used different methods and sources for data collection in order to have different perspectives, and we believe that this is a strong side of our study. However, we have not tried to analyze each of these materials separately.

All of the 13 GPs who were interviewed about their own patients had seen intervention patients as well as control patients. The answers from the intervention patients, given directly to and written down by the GPs, may have been biased in some cases, perhaps in a too positive direction. Some intervention patients may have found it difficult to tell their own GP about negative feelings concerning their cancer care. However, the questions did not focus specifically on the care given by the GP. We think that the three open questions favoured answers and themes that were of genuine concern to the patients.

There may have been some ambiguity concerning the time period in focus in this study. We think that most patients focussed on their present situation: few months after the end of treatment. GPs were interviewed 1–3 years after the diagnosis had been made and may have reflected upon a more prolonged period of follow-up, although the interviews focused on what happened to the patient during the first months after diagnosis and treatment. GPs in the focus group were asked to give examples of patients they had cared for and focused mainly on the time immediately before and after the cancer diagnosis was made, but some used examples on long time follow-up.

### The themes

We think that the five main themes that emerged from the different texts deal with two complementary ways of looking at primary care involvement in follow-up for patients with cancer: What kind of follow-up the patient wants, and what kind of follow-up the GP is able to offer.

#### A. What kind of follow-up does the patient want?

Follow-up of patients with cancer must face two kinds of reality: the type and stage of the disease, and the individuality of the patient. In our study there are few patients for each type of cancer, and we could not decide whether the anatomical location of the cancer decides the need for general practitioner assistance. This could be a topic for further study. Disease stage seemed important. Most of our patients had stable disease at the time of the study, and thus experienced little illness. If the initial hospital treatment is successful, many patients with cancer need to feel cured rather than talking to their GP about the cancer. We found that patients differed widely as to whether they wanted to talk much about their cancer and be fully informed and that the GPs fully acknowledged this (theme 1). We also found that the need to be treated as 'a whole person' is not the same for every patient (theme 2). These findings are in accordance with findings in other patients [11]. If the patient feels ill, a contact with the GP may be more valuable. Co-morbidity in the sense that the patient needs medical follow-up for other conditions in addition to the cancer is mentioned by some patients and adds to the illness-dimension [12]. Also, illness in other persons living with the patient may influence the need for care. Both patients and GPs mention the unique possibility in general practice to give comprehensive care. Information on diagnosis and future conditions, education about cancer, support groups and hospice referral are important patient concerns [13,14]. Transportation, lodging and questions about alternative medicine were also important topics. An earlier study had similar findings, and also considered patient finances [15]. Stigma has been studied in relation to lung cancer and found to be important [16].

When a GP collaborates with home services, many of these questions can be addressed in primary care [17]. Most patients with rapid disease progression soon after the end of treatment were excluded from our study. GPs do participate in palliative home care and may perform better if a co-operation with specialists is formalized [18]. After cancer treatment, it is common to feel a need for stimulating natural defence mechanisms in the body [19,20], for example through healthy eating or physical exercise, as suggested by one relative. Many patients also use various kinds of alternative or complementary medicine with such a goal in mind [21].

#### B. What kind of follow-up can the GP offer?

Several of the interviewed GPs mentioned their feeling of not knowing enough about cancer (theme 4). However, many GPs gave examples of shared care where they asked and obtained specialist advice when they needed it for the individual patient, rather than as part of a general teaching. These GPs often take considerable responsibility for medication and follow-up when patients want it. These findings support a systematic approach to shared care (theme 5). Shared care can promote a good dialogue between the GP and the expert professionals and give access to learning the most relevant practical procedures applicable to each patient [22,23]. For some major forms of cancer such as breast cancer and colorectal cancer, routine relapse detection can be delegated to a well-functioning primary care team [24,25] and can be combined with psychosocial assistance. In Norway, rural doctors sometimes undertake more extensive oncological treatment and follow-up in co-operation with the hospital oncologists in order to permit a patient to travel less and stay more at home. Such services in rural areas are of concern in other countries as well [26]. Asking for and acknowledging the expectations and preferences of each individual patient is a central aspect of a patient-centred working style [27] and strengthens empowerment of the patient [28,29]. Preservation of dignity is an overall aim and the GP may contribute to this [30]. Increased self esteem and empowerment are closely related to improved outcome for several diseases, cancer included [31].

The GP is busy, but has a kind of stability that rarely can be matched in a more complex system such as the hospital. Several patients in our study appreciated this (theme 3). The division of labour between primary and secondary care is more clear-cut during the early diagnostic and therapeutic phases of disease than for follow-up. Only a handful of GPs felt that they were part of a follow-up team early after the patient's treatment. Even when a GP makes frequent home visits to a terminally ill patient and actively contributes medically and psychologically during the last part of a patient's life, the hospital most often remains the formal co-ordinator [32]. Further research is needed in order to identify and overcome obstacles to a better shared care for patients with cancer.

## Conclusion

The GP has a place in the follow-up of patients with newly treated cancer, but hardly for all of them. Many patients with cancer need the continuity of care from one personal GP who knows them well, and trust the GP to provide competent care. Most GPs are prepared to take a more prominent role in the follow up for these patients and are aware of the need for individualized care. Formalized cooperation with a personal GP should be established for most patients with cancer before they leave the hospital. In this way competent advice could be available for the GP and his/her patient as is needed. Patients as well as GPs call on hospital doctors who are in charge of treatment to initiate such collaboration for individual patients.

## Competing interests

The author(s) declare that they have no competing interests.

## Authors' contributions

KH and TA developed the core idea, and all the authors were involved in the design of the study. KH raised the research funds and translated the 'Patients' Views of Cancer Services' questionnaire. TA and HM carried out the interviews with GPs. TA prepared the texts for the QSR-N6 analysis. All the authors read the text and participated in the analysis. TA wrote the first draft and the final manuscript and KH made important contributions to the manuscript. All authors read and approved the final manuscript.

## Pre-publication history

The pre-publication history for this paper can be accessed here:


